# Supplementary material for: Alliin, An Allium sativum Nutraceutical, Reduces Metaflammation Markers in DIO Mice
Source: Nutrients. 2020 Feb 27;12(3):624. doi: 10.3390/nu12030624 (PMC7146142; doi:10.3390/nu12030624)
Supplement: Supplementary file 1 [file nutrients-12-00624-s001.pdf]

Supplementary Table S1. Effect of diet HFD on metabolic tests.

	**STD**	**HFD**	***p***
Body weight (gr)	28,30 ± 2,077	40,52 ± 4,708	< 0.0001
Triglycerides (mg/dL)	103,7 ± 21,54	162,2 ± 40,76	< 0.0001
Cholesterol (mg/dL)	152,9 ± 2,562	159,4 ± 11,28	< 0.001
Fast glucose (mg/dL)	103,5 ± 17,62	126,6 ± 16,77	< 0.0001
AUC of OGTT (a.u.)	17023 ± 2872	21960 ± 5180	< 0.001
AUC of ITT (a.u.)	8944 ± 1680	14381 ± 2950	< 0.0001

The values are expressed as mean ± SD.
